# Bringing Barley Back: Analysis of Heritage Varieties for Use as Germplasm Sources to Improve Resistance against the Most Devastating, Contemporary Disease in Canada, Fusarium Head Blight (*Fusarium graminearum*)

**DOI:** 10.3390/plants13060799

**Published:** 2024-03-11

**Authors:** James R. Tucker, Ana Badea, Barbara A. Blackwell, Dan MacEachern, Aaron Mills

**Affiliations:** 1 Brandon Research and Development Centre, Agriculture and Agri-Food Canada, 2701 Grand Valley Road, Brandon, MB R7A 5Y3, Canada; ana.badea@agr.gc.ca; 2 Ottawa Research and Development Centre, Agriculture and Agri-Food Canada, 960 Carling Avenue, Ottawa, ON K1A 0C6, Canada; barbara.blackwell@agr.gc.ca; 3 Charlottetown Research and Development Centre, Agriculture and Agri-Food Canada, 440 University Avenue, Charlottetown, PE C1A 4N6, Canada; dan.maceachern@agr.gc.ca (D.M.); aaron.mills@agr.gc.ca (A.M.)

**Keywords:** fusarium head blight, deoxynivalenol, barley, germplasm resources

## Abstract

Fusarium head blight (FHB), caused by *Fusarium graminearum*, is currently the most devastating disease for barley (*Hordeum vulgare*) in Canada. Associated mycotoxins can compromise grain quality, where deoxynivalenol (DON) is considered particularly damaging due to its frequency of detection. Breeding barley with a lower DON content is difficult, due to the poor adaptation and malt quality of resistance sources. A set of European-derived heritage varieties were screened in an FHB nursery in Charlottetown, PE, with selections tested at Brandon, MB, between 2018–2022. Genetic evaluation demonstrated a distinct clustering of Canadian varieties from the heritage set. At Brandon, 72% of the heritage varieties ranked lower for DON content than did the moderately resistant Canadian check ‘AAC Goldman’, but resistance was associated with later heading and taller stature. In contrast with Canadian modern malting variety ‘AAC Synergy’, general deficiencies were observed in yield, enzyme activity, and extract, along with higher protein content. Nonetheless, several resistant varieties were identified with reasonable a heading date and yield, including ‘Chevallier Chile’, ‘Domen’, ‘Djugay’, ‘Hannchen’, ‘Heils Franken’, ‘Moravian Barley’, ‘Loosdorfer’ with ‘Golden Melon’, ‘Nutans Moskva’, and ‘Vellavia’, these being some of the most promising varieties when malting quality characteristics were also considered. These heritage resources could be used as parents in breeding to develop FHB-resistant malting barley varieties.

## 1. Introduction

Fusarium head blight caused by *Fusarium graminearum* Schwabe (teleomorph *Gibberella zeae* (Schw.) Petch) has long been known to be a pathogen of cereals, where it was first identified in England well over a century ago [1]. This disease may impact yield under severe epidemics but is primarily a quality concern due to the range of mycotoxins it can produce, including deoxynivalenol (DON). Deoxynivalenol is particularly problematic due to its common frequency of detection and requirement for regular monitoring by grain merchants, where grain lots >0.5 mg kg^−1^ are often rejected for sale into the lucrative malting industry. The economic importance of this disease of barley (*Hordeum vulgare* L.) for North America was elevated following several epidemics occurring during the mid-1990s in the Red River Valley [2]. Impacts of this disease on the North American malting barley industry are immense, with economic losses valued at several hundred-million dollars annually, and has contributed to reductions in total area seeded in favour of other crops [3]. Since the occurrence of the earlier epidemics, the disease has spread further west to occupy the major barley production regions of Canada. As in many other countries around the world, it is now considered in Canada to be the most devastating disease of barley. Canada is a significant producer of high-quality barley, where a large portion of its annual harvest is exported to supply the malt requirements of the world. 

Fungicides that provide the suppression of FHB in barley are available; however, they are costly and must be applied during a narrow window following spike emergence [4]. Furthermore, such control agents may impact off-target organisms and are not acceptable in organic production systems. Disease management through the use of varieties with inbuilt resistance is the most economically feasible and environmentally responsible approach; however, the quantitative nature of FHB resistance in barley makes this a challenging task. Immunity to FHB is not known in barley, and the availability of resistance sources is limited for use in breeding [5]. Unlike in other cereals such as wheat where major quantitative trait loci (QTL) have been identified, resistance in barley is conferred by many minor genes [6]. Coincidental agronomics (extreme heading; tall stature) of common resistance sources are most often undesirable. Moreover, these resistant materials generally do not possess satisfactory malting quality [7]. Crossing involving unimproved accessions has imposed restrictions for variety development, due to the numerous problems associated with linkage drag. Typically, several back-crosses have been required to achieve acceptable malting quality, with resultant varieties representing a compromise to full resistance expressed within donor parents.

European varieties first introduced into Canada served as an important foundation of modern varieties [8]. However, barley breeding in Canada began from a population bottleneck through the initial utilization of relatively few varieties [9]. Breeding efforts imposed to maximize production in response to the demands of the time may have resulted in further losses of genetic variation. Fusarium head blight has had a much longer history in England and other European countries than in Canada. The heritage landrace varieties used in this study are European-based and may have inadvertently been developed through selective seed-saving methods under pressures of FHB. Old-English malting variety ‘Chevallier’, which was a dominant variety from almost two centuries ago, has been identified in European environments with a high level of FHB resistance and a corresponding reduction in DON [10,11]. Furthermore, Canadian breeding programs have effectively used Swedish heritage variety ‘Svanhals’ as an FHB resistance source. This heritage variety has demonstrated resistance in the western Canadian environment [12,13], and has been useful for developing elite two-row malting barley germplasms such as TR05287 (Svanhals/AC Metcalfe//TR253 [14]). This study had the purpose of evaluating a set of historic European varieties for their value as germplasm sources of resistance to FHB and DON accumulation, as tested under Canadian growing conditions. Heritage barley varieties are historically improved germplasms that may represent practical sources of resistance for the development of modern, resistant varieties with less limitations than those of varieties arising from alternate exotic sources.

## 2. Results

### 2.1. Fusarium Head Blight Nursery Evaluation

An analysis of the FHB index and DON content of the whole heritage barley variety set (*n* = 80), assessed at Charlottetown, PE, demonstrated a wide range of infection, as measured using the FHB visual index (3.3–49.0%) and DON content (8.0–87.7 mg kg^−1^). Deoxynivalenol content was not significantly correlated (P > 0.05) with any measure of FHB visual symptoms, possessing no relationship with incidence (r = −0.02) and a weak relationship with severity (r = 0.13). A selection of 38 varieties was made for further evaluation in western Canada, based primarily on the ranking of mean DON content (Supplementary Table S1). Interestingly, ‘Chevron’ and ‘Svanhals’ were not selected for advanced study from the initial test. 

The selected group of varieties were evaluated at Brandon, MB, over a five-year period (2018–2022). Disease pressure varied by year, as indicated by the mean FHB rating and DON content (Table 1). Weather patterns varied between the locations (Charlottetown vs. Brandon) and within Brandon over years (Supplementary Table S2), where warmer temperatures, particularly during August under the prolific growth stage, were associated with higher disease and DON content in grains. In all years, mean DON content was quantified above the Canadian limit for the selection of grains for sale to the malting industry (<0.05 mg kg^−1^). Of all heritage varieties evaluated, only 15% and 28% ranked higher than cultivars ‘AAC Synergy’ (intermediate) and ‘AAC Goldman’ (moderately-resistant), respectively, for DON content. All heritage varieties that accumulated greater DON content than two-row check ‘AAC Synergy’ were six-row typed. Several heritage varieties accumulated less than half of the DON content of that of ‘AAC Synergy’, which was in the typical range of the best-known sources of resistance that have been commonly used by barley breeders. 

A test of normality for DON content indicated that the distribution was right-skewed (Shapiro–Wilk W = 0.90, P < W = 0.001), and a logarithmic transformation was applied in all further analyses. Least squares variety means for FHB ratings and DON content are presented in Table 2. Heritage varieties with the lowest FHB symptoms included ‘Kitchin’, ‘Hakata 2’, ‘Loosdorfer’, ‘Chevallier 1’, ‘Chevallier Chile’, ‘Golden Pheasant’, and ‘Long Eared Nottingham’. The two-row variety ‘Prior’ had the highest overall symptoms, exceeding all of those of the six-rowed varieties, which were generally susceptible to FHB as a group. The heritage varieties with the lowest DON content were the two-row varieties ‘Domen’, ‘Heils Franken’, ‘Loosdorfer’, ‘Proctor’, ‘Golden Melon’, ‘Long Eared Nottingham’, ‘Hannchen’, ‘Vellavia’, and ‘Chevallier French’. Alternately, six-row type varieties displayed the highest DON content of the group; nonetheless, pigmented and hulless varieties showed relatively low DON content within this class. While the correlation for DON for the sub-set between the Charlottetown, PE, and Brandon, MB, sites was moderate (r = 0.19), the group of best-ranked varieties were commonly identified at either location.

**Figure 1:**
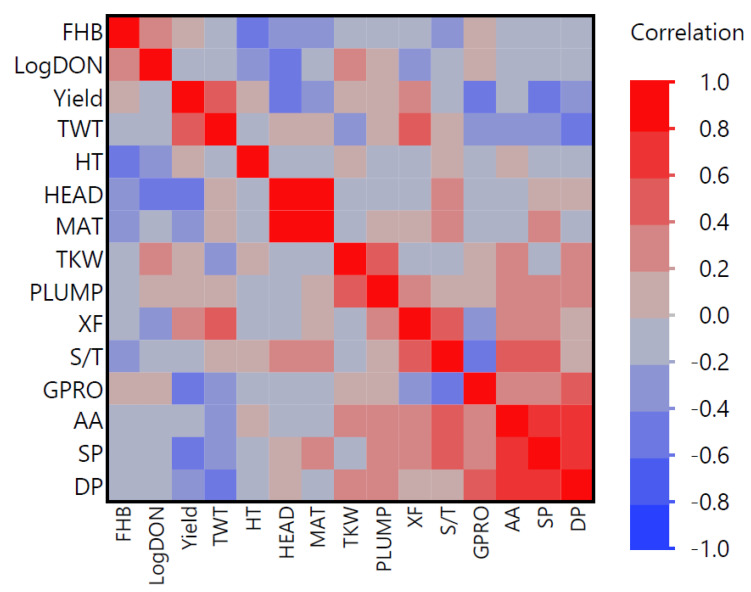
Heat map of Pearson’s correlation coefficients between characters: Fusarium head blight rating (FHB); Log_10_ deoxynivalenol content (LogDON); yield (kg ha^−1^); test weight (TWT); height in cm (HT); days to heading (HEAD); days to maturity (MAT); thousand kernel weight (TKW); percent plump kernels (PLUMP); fine grind extract (XF); soluble to total protein (S/T); grain protein (GPRO); alpha amylase (AA); soluble protein (SP); diastatic power (DP).

Averaged over years at Brandon, FHB ratings demonstrated a moderate, positive association with Log_10_ DON content (r = 0.37, P = 0.03) (Figure 1). Fusarium head blight ratings and Log_10_ DON, respectively, were negatively associated with height (r = −0.56 and −0.34, and P = 0.0005 and P = 0.046, respectively). Log_10_ DON was negatively associated with days to heading (r = −0.44, P < 0.01). Fusarium head blight and Log_10_ DON content were not associated with any of the malting quality characteristics (P > 0.05). 

### 2.2. Agronomic Characteristics

The set of heritage varieties displayed a wide range for heading days, both in yield-plots (18.6 days) and the FHB nursery plots (15.8 days), with the majority of heritage varieties heading later than the modern varieties (Table 3). Earlier-heading varieties were generally of the six-row type; however, two-row varieties such as ‘Prior’ and ‘Scotch Common’ also headed very early. While plots headed somewhat earlier in the non-irrigated yield plots (P < 0.0001), a robust correlation was observed in the days to heading with the FHB nursery plots (r = 0.92, P < 0.0001). Days to heading was correlated with days to maturity (r = 0.83, P < 0.0001). Plots grew >15 cm taller under nursery irrigation, and nursery height displayed a moderate correlation with yield plots (r = 0.67, P < 0.0001). Certain varieties, such as semi-dwarf ‘Golden Promise’ and pigmented six-row variety ‘Mestny’, grew proportionately taller in the FHB nursery. While the average height of the heritage variety set was similar to the average height of the modern varieties, there was a considerable range between them (26.0 cm). Yield was negatively associated with days to heading (r = −0.43, P = 0.01) and days to maturity (r = −0.35, P = 0.04). 

On average, the heritage varieties yielded 2425 ± 61.5 kg ha^−1^, which was 79% of the mean yield of Canadian modern check varieties. As might be expected, a number of European heritage varieties were poorly adapted in the Canadian environment, showing high yield deficiencies in contrast with the Canadian modern varieties, i.e., two-row varieties ‘Ducksbill’, ‘Golden Promise’, and ‘Golden Pheasant’, along with six-row varieties ‘Sativum Jessen England’ (pigmented) and ‘Bohmische Nackte’ (naked). Conversely, varieties such as ‘Larker’ (six-row), ‘Domen’, ‘Nutans Moskva’, ‘Isaria’, ‘Hannchen’, and ‘Vellavia’ displayed notable yields, which were comparable to those of Canadian modern varieties.

### 2.3. Physical Grain Characteristics 

On average, test weights of the heritage varieties were near the levels of those of Canadian modern varieties (Table 4). In fact, a number of the heritage varieties exceeded the Canadian modern varieties including ‘Isaria’, ‘Nutans Moskva’, ‘Gotlands’, ‘Ketch’, and ‘Chevallier French’. Alternatively, the heritage varieties generally demonstrated greater deficiencies in their thousand kernel weights and their percent of plump kernels, averaging 86.1% and 90.0% of Canadian modern variety means, respectively. Most of the six-row heritage varieties evaluated displayed a lower thousand kernel weight and plump percentage, with the exception of ‘Larker’, an American variety specifically bred for improved kernel plumpness. Two-row heritage varieties demonstrating the highest thousand kernel weight and kernel plumpness included ‘Hakata 2’, ‘Heils Franken’, and ‘Kitchin’. Percent plump was positively associated with thousand kernel weight (r = 0.45, P < 0.001). Test weight was positively associated with yield (r = 0.48, P = 0.004).

### 2.4. Malt Quality

Generally speaking, the optimal range of grain protein for malting purposes falls within 11.0–12.5% and is a major determinant of grain suitability according to maltsters to meet needs of the breweries. On average, grain protein levels in 2021 (14.7 ± 0.2%) were much higher than they were in 2018 (12.4 ± 0.2%) and significantly different (t = 2.0, P < 0.0001). Accompanying this was soluble protein concentration, which also displayed a significantly higher (t = 2.00, P < 0.0001) level in 2021 (4.26 ± 0.06%) compared with 2018 (4.92 ± 0.06%). All other malt quality characteristics measured did not differ significantly between years (P > 0.05).

Grain protein was negatively associated with test weight (r = −0.36, P = 0.03) and yield (r = −0.39, P = 0.02). Grain protein was positively associated with soluble protein (r = 0.40, P = 0.02) and diastatic power (r = 0.54, P = 0.0009), and negatively associated with soluble to total protein (r = −0.52, P = 0.002) and yield (r = −0.52, P = 0.002). Fine grind extract was positively associated with yield (r = 0.40, P = 0.02), alpha amylase (r = 0.39, P = 0.004), and soluble to total protein (r = 0.50, P = 0.003). Alpha amylase was positively associated with diastatic power (r = 0.71, P < 0.0001), soluble protein (r = 0.77, P < 0.0001), and soluble to total protein (r = 0.47, P = 0.005). 

Overall, none of the heritage varieties displayed malting quality profiles aligned to the standards of Canadian malting barley (Table 5). However, there was a considerable range within the set for the malting quality characters assessed, where several varieties were much closer to the quality expectations set by the malting industry. All of heritage varieties possessed higher levels of grain protein than did ‘CDC Copeland’ or ‘AAC Synergy’, with several varieties displaying extremely high levels. Several heritage varieties had protein levels equal to/ lower than those of ‘AC Metcalfe’ including ‘Bere’, ‘Chevallier 1’, ‘Golden Melon’, ‘Hen Gymro’, ‘Isaria’, ‘Scotch Annat’, and ‘Nutans Moskva’. Varieties such as ‘Bere’, ‘Isaria’, and ‘Scotch Annat’ had low grain protein, yet they also had low soluble to total protein values. A number of other heritage varieties had moderate protein levels below that of ‘AAC Ling’, which is grown for livestock feed. The heritage variety set generally possessed lower fine extract than did ‘AAC Synergy’, CDC Copeland’, or ‘AC Metcalfe’; however, several displayed notably higher levels, including ‘Chevallier French’, ‘Golden Melon’, ‘Kneifel’, ‘Loosdorfer’, ‘Nutans Moskva’, and ‘Vellavia’.

High enzymatic activity is required for a brewing process that uses adjuncts, while lower enzymatic activity is required for all-malt craft beer production with no or limited adjuncts. None of the heritage varieties rivalled the alpha amylase activity of ‘AC Metcalfe’, ‘CDC Copeland’, or ‘AAC Synergy. Varieties with higher alpha amylase activity approaching that of the modern Canadian varieties included ‘Ducksbill’, ‘Golden Pheasant’, ‘Scotch Common’, and ‘Vellavia’. Several varieties, ‘Ducksbill’, ‘Golden Promise’, ‘Hakata 2’, and ‘Scotch Common’, displayed high diastatic power above the levels of that of ‘CDC Copeland’ and ‘AAC Synergy’, nearing that of ‘AC Metcalfe’. The heritage variety set generally possessed lower fine grind extract than did the Canadian malting cultivars. 

### 2.5. Genetic Diversity

**Figure 2:**
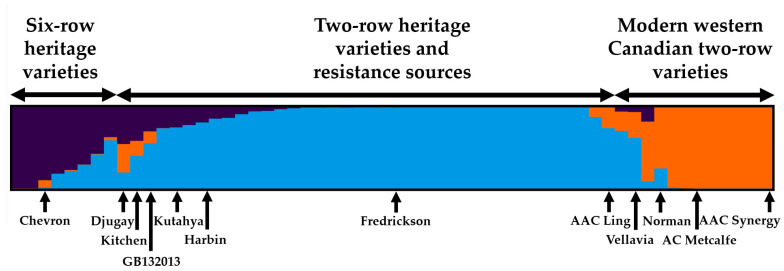
Sub-population membership for 58 barley genotypes grouped by three major clusters of genetic variants including (1) six-row varieties (purple); (2) Modern Canadian varieties (orange); (3) Two-row heritage and resistance source varieties (blue).

Genetic diversity was assessed for the heritage barley set, modern Canadian germplasm, and exotic resistance sources that were used for the improvement and development of FHB-resistant varieties. An analysis of population structure indicated a probability of three (K = 3) major clusters (Figure 2). The first cluster (15% overall membership) mainly included six-row heritage varieties, of which Swiss resistance source ‘Chevron’ was a member. Several heritage two-row varieties such as ‘Djugay’, ‘Kitchen’, and ‘Prior’ held significant membership at 26–45% in this cluster. Resistance sources ‘GB132013’, ‘Kutahya’, and ‘Harbin’ also displayed partial membership, at 22–30%. The second major cluster (20% overall membership) involved all modern Canadian varieties besides ‘Island’. The largest cluster (65% overall membership) held all two-row heritage barley varieties, along with common FHB resistance sources. This two-row heritage group generally did not show much admixture with group 2, with the exception of some varieties such as ‘Djugay’, ‘Ducksbill’, ‘Kitchen’, and ‘Vellavia’. 

**Figure 3:**
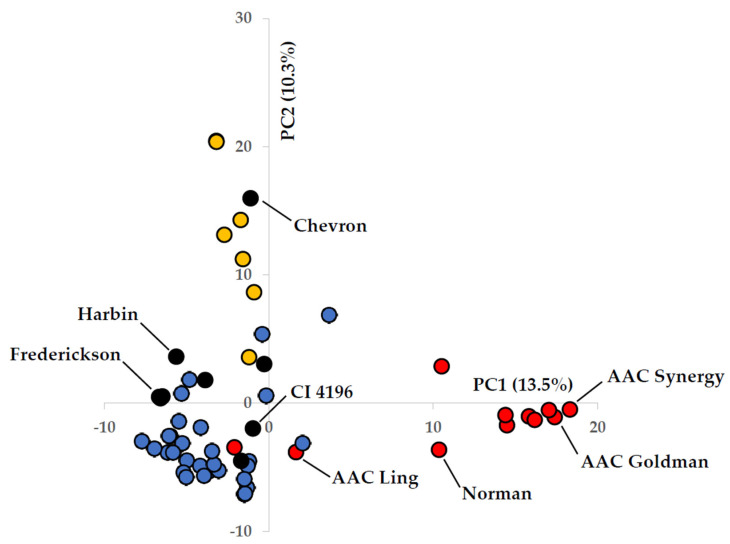
Scatter plot of analysis of first two principal components of genotype information involving 2358 single-nucleotide polymorphic (SNP) markers with colour highlights: (1) yellow points represent six-row heritage varieties; (2) blue points represent two-row heritage varieties; (3) black points represent common FHB resistance sources; (4) red points represent modern Canadian varieties.

Western Canadian varieties were less genetically diverse, displaying closer relationships amongst themselves, and were collectively distinct from the European heritage variety groups. Of all the heritage varieties evaluated, French variety ‘Vellavia’ demonstrated the closest relationship with the modern western Canadian varieties. The two eastern Canadian varieties included in the study, ‘AAC Ling’ and ‘Island’, were inter-related to each other and to Swedish resistance source ‘Svanhals’ (Supplementary Figure S1). The eastern Canadian varieties demonstrated less divergence from the heritage varieties in comparison with the western Canadian variety group (Figure 3; Supplementary Table S3). Two-row resistance sources included in the study generally originated from China, including ‘Frederickson’, ‘Russian 6’, ‘Zhedar 1’, ‘Harbin’, and ‘CIho 4196’, which demonstrated kinship with each other. To some degree, they shared a genetic relationship with certain varieties such as ‘Ducksbill’. Resistance sources ‘Kutahya’ and ‘GB132013’ appeared somewhat distinct from the other sources originating from China. For example, ‘GB132013’ was derived from a complex cross involving six-row variety ‘OAC Kippen’ and a line with ‘Proctor’ in pedigree (Dr. Duane Falk, personal communication). While the resistance sources that were included for general comparison shared the same major cluster group, they did not show strong kinship relations with the heritage variety set. 

## 3. Discussion

Germplasm collections offer a means to evaluate phenotypic and genetic data for panels of materials representative of progress in breeding from across historical periods in contrast to that of modern-day varieties [17]. This study has demonstrated that significant opportunities lie in wait for the exploitation of genetic variance identified for resistance to FHB and DON accumulation in the assessed set of European heritage barley varieties. Maritime climatic conditions at Charlottetown site can be very conducive to disease development, due to warm temperatures and high humidity. Consequently, this environment provided high disease load and resulting strong selective pressure for the identification of resistant genotypes from the preliminary set. While there is no immunity and/or qualitative resistance (R genes) documented for this disease in barley, these heritage varieties may carry genetic factors that may contribute to partial resistance. Fusarium head blight evaluations in barley are challenging due to the high variations associated with infection patterns and the specialized chemistry requirements for measuring minute levels of mycotoxins, yet this process proved to be an effective mechanism with which to identify resistance sources. The majority of heritage varieties selected from the initial nursery evaluation at Charlottetown, PE, and re-tested at Brandon, MB, over a five-year period consistently displayed levels of resistance comparable to those of some of the best-known resistance sources extensively used by breeders for crossing such as ‘Chevron’, ‘Harbin’, ‘Fredrickson’, and ‘Zhedar 1’. Even the most reliable FHB resistance sources of barley will suffer some degree of infection and will produce measurable levels of DON. Several of the Chinese-based resistance sources commonly used in the past by barley breeders demonstrated high levels of inter-relatedness in the current work, as was documented in previous study [18,19]. This study documents several heritage varieties that display low DON production, significantly below that of modern varieties, where underlying partial resistance factors may be very useful to barley breeders seeking to develop new resistant varieties. The exploration of genetic variation of European landrace varieties has successfully identified novel resistance to *Rhynchosporium commune*, the barley pathogen responsible for scald disease [20]. Beyond the identification of FHB resistance, the set was further characterized for agronomic potential and malting quality, to identify the most suitable parents for new crosses. 

The evaluation of the genome-wide genetic variation (multi-locus SNP markers) of the heritage barley set in this study revealed that the modern western Canadian elite malting germplasm demonstrated a genetic distinction from the heritage varieties. Furthermore, the heritage varieties evaluated in the study did not demonstrate close relationships to Chinese-based exotic resistant varieties commonly used by breeders. Given the history of barley breeding in Canada, some of the heritage varieties used in this study may have contributed genetics to this germplasm based on foundational crosses. Through selection over time, improvements have been made in agronomics in combination with enhancements in malting quality to develop the high-quality products that Canada is recognized for producing. However, additive genetic variance has been eroded through strong selection pressure towards ideotype malting varieties that provide consistency to the malting industry. The breeding of malting varieties is complicated by the extremely strict guidelines set by the industry, where one single deficiency among the many characters evaluated will result in the rejection of a breeder line from being supported for registration. Genetic blocks of variation lost in historical breeding efforts may have been the result of selections imposed in an environment where Fusarium damage was a minor concern. While *F. graminearum* has been documented within Canada for a long period, it only became of economic concern following epidemic years in the mid-1990s [2]. Currently, this is the most devastating disease of barley in North America, amounting to economic losses of billions of dollars [21]. As a result, FHB resistance and low DON accumulation are top-priority breeding targets set by the malting and brewing industries.

While resistance to DON production is critically important to barley production, breeders are faced with the challenges of developing varieties with suitable agronomics that can compete with other commodities that can be grown on the farm. Almost half of the heritage varieties displayed an FHB rating lower than that of ‘AAC Synergy’. Heritage varieties with the lowest FHB symptoms such as ‘Kitchin’, ‘Hakata 2’, ‘Chevallier 1’, ‘Chevallier Chile’, ‘Loosdorfer’, ‘Golden Pheasant’, and ‘Long Eared Nottingham’ were also the tallest of all entries tested. Likewise, most of the aforementioned group of varieties showed a tendency to head out late. From the general set, several FHB-resistant varieties were identified as ‘Chevallier’-types or progeny varieties, implying that they may carry related, heritable variation. However, as documented by Hagenblad and Leino [22], commonly classified ‘Chevalier’ varieties may be in fact genetically distinct as a result of the growth in mixtures, resulting in spontaneous outbreeding and hybridization. For example, they define ‘Chevallier French’ and reputed Chevallier selection ‘Scotch Common’, included in this study, as non-true Chevallier-types in comparison with museum specimens. Due to extreme heading/ height, it is not completely possible to say if the nature of resistance in extreme varieties is antibiotic or simply an escape of infection through spike extensions above the canopy, which increases drying through wind or late heading that avoids heightened periods of rainfall. The Australian heritage variety ‘Prior’ (PI-67315) is believed to be a farmer selection from ‘Chevallier’, but also English variety ‘Archer’ is recognized as a potential parent. In the current study, ‘Prior’ was one of the earliest varieties standing 14 cm shorter than ‘Chevallier 1’ and achieved the highest FHB score of the study (FHB = 4.0). While ‘Prior’ had the highest overall infection level, it exhibited moderate DON levels. While ‘Chevallier’ types are known to be tall and late-heading, quantitative trait loci (QTL) for resistance have been identified for FHB without a linkage to negative agronomic traits [11]. While barley universally carries type II resistance, i.e., the prevention of spread through the rachis node from the initial point of infection [14,23] because of the ubiquitous expression of UDP-glucosyltransferase detoxification genes, ‘Chevallier’-types may also carry additional resistance to external disease spread as antifungal metabolites that limit external hyphae growth and/or morphological resistance features associated with trichomes, husk thickness, and/or grain hardness [10]. The American variety ‘Kitchin’, which displayed low FHB in this study, was previously identified as a source of resistance [24] and used for crossing in breeding programs. 

Fusarium head blight displayed a moderate association with DON in this study; however, the heritage varieties with lowest FHB ratings were not necessarily the ones with the lowest DON content. Some Fusarium-resistant varieties such as ‘Loosdorfer’ also accumulated low DON. Yet, the sub-group of varieties with the lowest DON including ‘Domen’, Vellavia’, ‘Proctor’, ‘Heils Franken’, ‘Golden Melon’, and ‘Hannchen’ all displayed some degree of infection. These varieties showed slightly elevated FHB symptoms compared with the aforementioned group, but generally had more reasonable stature heading dates. Heading was also closer to that of Canadian checks; however, later heading occurred in varieties as ‘Proctor’ and ‘Hen Gymro’. Several of such varieties exhibiting low DON characteristics demonstrated some level of kinship (Supplementary Figure S1). Relationships between FHB and DON are complex in barley, where resistances to either character may be independent or even oppositional in some cases. The pathogen is considered hemi-biotrophic and switches from biotrophy to necrotrophy at approximately 72 h post-infection, where Fusarium-susceptible parents may also contribute resistance alleles for low DON [25]. Numerous bi-parental studies conducted to identify FHB resistance in barley have shown that QTLs associated with FHB resistance are not always coincident with those for DON accumulation [6]. Resistance to this disease of barley may be somewhat bipartisan, associated with mechanisms of infection, and/or a reduction in mycotoxin accumulation. Deoxynivalenol is the product of FHB that is used as a biomarker by grain merchants to determine quality of grain. Fusarium head blight resistance may be achieved through various mechanisms; nevertheless, they must collectively contribute to lowered DON accumulation.

Nordic variety ‘Domen’ has been previously identified with low DON characteristics [26]. This variety was derived from cross Opal B/Maskin, where the ‘Opal’ pedigree is Binder/Gull, with parental descendance from ‘Hanna’ and ‘Gotlands’. Likewise, ‘Kenia’ and ‘Maja’ also share the pedigree Binder/Gull, where ‘Maja’ is a common source of resistance [27] and ‘Kenia’ is a parent of FHB-resistant ‘Proctor’ (as per this study). In the Canadian context, ‘Domen’ is a parent of the moderately resistant variety ‘Klages’ [28] and a grandparent of the two-row malting cultivar ‘Harrington’ [29]. ‘Harrington’ is an important variety that helped establish the two-row barley market as the dominant malt barley kind produced in Canada for over two decades, which has been used by American breeding programs as a resistance source. ‘Loosdorfer’ and ‘Hannchen’ are both selections from theMoravian-style variety ‘Hanna’, where ‘Hannchen’ was also reported to have low DON when infected by *F. culmorum* [10]. ‘Hannchen’ was grown as a malting variety in Canada, assessed as having “relatively high yields in certain parts of western Canada” [30] and noted to be better adapted to higher-moisture conditions [31]. It was used as a parent in crosses at the University of Saskatchewan, producing varieties such as ‘Rex’ (CI-6618). In a previous study by Muhammed, 2012 [10], the French variety ‘Vellavia’ demonstrated higher Fusarium susceptibility within UK trials than those observed under Canadian growing conditions, which demonstrates the need to evaluate germplasm in environments intended for production. 

While productivity potentials measured through yield were lacking in several of the heritage varieties, kernel size as measured through thousand kernel weight and percent plumpness was satisfactory for several heritage varieties. Prior to advanced malting chemistries, many varieties studied herein had been selected and used for malting purpose historically, where plump kernels were considered a desirable characteristic. Grain protein levels in the European heritage set were generally higher than those in the modern Canadian malting varieties; however, a range was observed within the set where several demonstrated acceptable limits, i.e., <12.5%, particularly in the more favourable 2018 growing conditions. In 2021, the dry and hot growing conditions experienced in western Canada contributed to much higher levels of protein in barley grain than normally observed. An average value of 13.2% was reported in 2021 for western Canadian barley, which was higher than the average level of barley proteins of 11.9% reported for 2018 [32,33]. Similar results were observed in Michigan, USA [34], where researchers evaluated a similar set of varieties and observed reasonable yield and kernel plumpness, and where they also found very high protein levels in certain varieties such as ‘Djugay’, ‘Ducksbill’, ‘Nurnberg’, and ‘Prior’. As in the current study, they documented lower grain protein in ‘Bere’ and ‘Chevallier 1’ varieties. In Brandon, alpha-amylase activity and diastatic power within the set was lower than ‘AC Metcalfe’, a high-enzyme variety tailored for use in adjunct brewing. A sub-group of heritage varieties demonstrated adequate enzyme activities, particularly for use in craft (all-malt) brewing. Likewise, several heritage varieties showed similar fine grind extract comparable to that of ‘AAC Synergy’. Goddard et al., 2019, compared ‘Chevallier’ with modern variety ‘NCF Tipple’ and found limits of alpha amylase activity, as was apparent in the current study [35]. Finding no further malt failings, they concluded that advancements in modern varieties were mainly attributable to improved agronomics vs. malting quality. Malting quality parameters of the heritage set evaluated in this study were inferior to those of modern varieties, but when combined with the value of low DON characteristics, several varieties displayed good potential for crosses to develop resistance. Benefits may be achieved through directed bi-parental genetic studies conducted to further investigate genomic regions of interest and identify QTLs for selective breeding. 

The foods of today are a much different version of what was produced and consumed by our forbearers. Human interventions, through the selection of crop characteristics, have resulted in significant improvements that have increased harvest index and provided the profitable production of barley. Germplasm collections of heritage varieties provide means to re-gain additive genetic variations for important characteristics such as disease resistance. The development of FHB resistance within modern malt varieties is an essential component of disease management and sustainable production with the benefit of reduced reliance on pesticides. Additive genetic variance is the foundation of inheritance for this disease, but is also a main determinate of plasticity through its interaction with the environment. Fusarium head blight commonly demonstrates GxE interaction in barley, and a flexible resistance response within the host may become more essential as the climate undergoes changes. The infusion of genetic variation may not only be useful for reductions in DON but may increase the range of other economically important characters. Resistant heritage barley varieties identified through this study provide hope for the future of barley production through protection against this devastating disease.

## 4. Materials and Methods

### 4.1. Fusarium Head Blight Nursery Phenotyping

Seeds of 80 heritage spring habit barley varieties were obtained from the Germplasm Resources Unit (GRU) at the John Innes Centre (JIC), Norwich, UK (via Dr. Chris Ridout & Dr. Sarah de Vos). In 2017, lines were sown in an irrigated FHB nursery at Charlottetown, PE (Harrington Research Farm, Latitude: 46°20′41.412″ N; Longitude: 63°09′53.246″ W), under replication in a randomized complete block design (RCBD, *n* = 3). In total 5–6 mL of barley seeds were planted in a 0.9 m row. Four locally sourced 3ADON *F. graminearum* isolates were grown in carboxylmethyl cellulose liquid media and mixed in equal proportions. After 75% of spikes had emerged, a macroconidia suspension with a concentration of 50,000 spores mL^−1^ was produced by mixing a 5 L concentrate within 250 L of water and applied to spikes using a CO_2_-powered researcher sprayer with 6 m boom and fan nozzles over a total area of 0.2–0.3 ha. The irrigation system supplied mist for a one-minute period in intervals of 15 min during daylight and every 60 min throughout the night. Visual symptoms were recorded on ten randomly selected spikes as percent (%) of infected kernels/spike (severity). Likewise, the percentage of infected spikes was recorded per row (incidence). From this, an index was calculated: incidence x severity/100. Matured grains were threshed using a stationary combine. A 20 g subsample was ground, and from this a 1 g representative sub-sample was collected. Deoxynivalenol content was measured using the in-house enzyme linked-immunosorbent assay (ELISA) technique at Agriculture and Agri-Food Canada, Ottawa Research and Development Centre, per Sinha et al., 1995 [36]. The most resistant material was selected, primarily based on the lowest DON content, for further testing (Table 6).

In 2018, the 38 varieties selected from the Charlottetown, PE, environment were planted in an FHB nursery at Brandon, MB (Brandon Research and Development Centre Latitude: 49°52′18.405″ N; Longitude: 99°58′23.673″ W) and evaluated in a RCBD experiment (*n* = 2). Plots consisted of a 0.9 m row sown with 30–40 kernels. A set of repeated Canadian FHB checks was planted before every 50 plots, including ‘AC Metcalfe’ (intermediate), ‘CDC Mindon’ (moderately resistant), and ‘CDC Bold’ (susceptible). Canadian two-row modern malting ‘AAC Synergy’, ‘Norman’, and ‘AAC Goldman’, and general purpose ‘AAC Ling’ cultivars were included as experimental entries. Grain spawn (maize kernels infected with two monoclonal isolates each of 15ADON (WRS1915 (NRRL 43162); WRS1918 (NRRL 43165)) and 3ADON (WRS2065 (NRRL 43217); WRS2067 (NRRL 43216)), chemotypes of *F. graminearum*) were spread on the soil surface weekly, for three applications in total. A fine-droplet irrigation spray was applied via sprinkler heads for a two-hour period at 6:00 a.m. and 6:00 p.m. Days to heading was recorded as the date when 50% of spikes emerged from the plot minus the sowing date. Height (cm) was recorded in 2022, as distance from the soil surface to the top of spike, excluding awns and averaged in measurements of the front and end of the plot. FHB ratings (0–5) were conducted 3.0–3.5 weeks after heading, as described in Tucker et al. 2022 [28]. Deoxynivalenol content was measured via ELISA, as previously detailed. This testing and the measurements were conducted and recorded for five years in total between 2018 and 2022, except for FHB ratings and days to heading, which were not recorded for 2018 and 2021, respectively. 

### 4.2. Agronomic and Malting Quality Evaluation

In 2018, due to seed limitations, micro-plots (double rows, 4.3 m long) were planted in the field at Brandon, MB, but at a distance to the FHB nursery. Canadian modern varieties ‘AC Metcalfe’, ‘AAC Synergy’, ‘AAC Ling’, and ‘CDC Copeland’ were included as agronomic check entries. Agronomic data were recorded for days to heading, height, and days to maturity. In 2021, the heritage varieties and checks were grown under replication (*n* = 3) in yield plots (six rows, 4.3 m long). Yields were adjusted to kg ha^−1^. Physical grain characteristics were recorded for each entry on a composite sample of the plots, including test weight (the density of grains expressed in kilograms per hectoliter (kg hL^−1^)), thousand kernel weight (the weight, in grams (g), of 1000 grains), kernel plumpness (percent (%) of grains retained when placed over a No. 6 - 6/64 in (2.38 mm) slotted screen). 

### 4.3. Malt Quality Analysis

Matured grains from 2018 and 2021 harvests were cleaned by using a SLN3 sample cleaner (Pfeuffer GmbH, Kitzingen, Germany) to remove chaff and debris. Canadian two-row malting varieties ‘AC Metcalfe’, ‘AAC Synergy’, and ‘CDC Copeland’ were included as malting quality check entries, and the two-row general purpose ‘AAC Ling’ was included as the non-malting check. Malting and malting analyses were conducted in Agriculture and Agri-Food Canada, Cereal Quality Laboratory at Winnipeg, MB. Malt was prepared using a custom-designed, automated micro-malting system. The malting process took place over one week using a routinely used specific regime. Several grain, malt and wort parameters were determined: grain protein concentration, fine grind extract, alpha-amylase activity, diastatic power, soluble protein concentration, and ratio of soluble to total protein concentration. Barley protein content (expressed as a percentage) was determined using FOSS Infratec ^TM^ 1241 Grain Analyzer (FOSS in North America, Eden Prairie, MN, USA). Fine-grind malt was prepared using Bühler Laboratory Disc Mill DLFU (Bühler Holding AG, Uzwil, Switzerland) on setting “2”. Fine extracts (expressed as a percentage) were prepared using an in-house-built, custom-designed extracting bath set at 67 °C. In total, 10 g of ground sample was extracted with 100 mL of water for 40 min, stirring it for 20 min, left to sit for 20 min, and then filtered with Whatman 4, 11 cm filter paper. Specific gravities were determined on the extract at 24 °C with Mettler Toledo DM45 Density Meter (Mettler Toledo, Columbus, OH, USA) calibrated with glucose standards. For enzyme activity, wort was prepared in an in-house-built, custom-designed extracting bath with room-temperature water. Briefly, 5 g of ground malt and 100 mL of calcium acetate were stirred for 20 min and filtered with Whatman 4, 11 cm filter paper. Alpha-amylase activity (expressed as dextrinizing units) and diastatic power (expressed as degrees Lintner) were determined with SEAL AA3 Continuous Segmented Flow Analyzer (SEAL Analytical Inc., Mequon, WI, USA) using glucose standards as calibrants and dextrinized ASBC starch as the substrate. Wort-soluble protein was determined spectrophotometrically on these samples as a secondary test.

### 4.4. Statistical Analysis

The distribution of DON content was evaluated for skewness, then subsequently transformed in Microsoft Excel using the function Log_10_ DON = Log_10_ (DON + 1). Statistical models were fitted in SAS JMP software v 17.2.0 (SAS Institute, Cary, NC, USA), where varieties were considered fixed effects, and then year and block were considered random effects. Least square means were calculated for various traits. Following this, all pairwise comparisons were constructed with Tukey’s honestly significant difference (HSD) test at a α = 0.05 probability level. Alternatively, DON was analyzed through the ranking of DON content varieties within replicate blocks, followed by the calculation of average rank. Pearson’s correlation coefficients between characters were also calculated using SAS JMP v 17.2.0 software. Characters were evaluated for normality via a Shapiro–Wilk goodness-of-fit-test (Prob < W). Three heritage varieties with unique characters (pigmented; hulless) were excluded from multi-character correlation analysis. 

### 4.5. Genetic Diversity Analysis

In addition to the European heritage barley set (38), several barley varieties (20) were included in the genetic diversity assay. These involved Canadian varieties like ‘AAC Synergy’, ‘AAC Ling’, ‘AC Metcalfe’, and ‘TR253’ along with those classified as moderately resistant to FHB, including ‘AAC Goldman’, ‘TR04282’, ‘AC Oxbow’, ‘Norman’, ‘CDC Mindon’, ‘Conlon’, and ‘Island’. Additionally, several Fusarium-resistant accessions commonly used by the barley breeding community were included such as ‘Chevron’ (6-row) and two-row barleys ‘CIho 4196’, ‘Harbin’, ‘Russian 6’, ‘Frederickson’ [27], and ‘Zhedar 1’ [7], along with ‘Kutahya’ and ‘GB132013’, which have been more recently identified within the FHB nursery at Brandon, MB, Canada.

Seeds were germinated on moist cotton balls in a seed starter tray under ambient light and temperature. At the two-leaf stage, tissue was sampled and freeze-dried. Following the protocol of Qiagen 96 DNeasy Plant kits (Qiagen GmbH, Hilden, Germany), DNA was extracted and evaluated for quality determination using NanoPhotometer N120 (Implen GmbH, Munich, Germany), and then diluted to 20 ng/uL in 10 uL. The DNA was sent to the Genomic Analysis Platform of the Institute of Integrative and Systems Biology (B. Boyle, Université Laval, Québec City, QC, Canada) for Genotype-by-Sequencing (GBS) library preparation using a two-enzyme system (one infrequent genomic DNA cutter, *Pst*I, in combination with a frequent genomic DNA cutter, *Msp*I [37]). Libraries were sequenced on Illumina NovaSeq 6000 S4, PE 150 format (2 × 150 bp reads), at the Génome Québec Center of Expertise and Services (Montréal, QC, Canada). 

The data processing of FASTA read sequence files was executed via the Fast-GBS v2.0 pipeline [38] using default settings. This pipeline uses various software including ‘Cutadapt v1.8.2’ for read trimming, ‘BWA v07.17’ for alignment, ‘Platypus v0.1.15’ for variant calling, and ‘BEAGLE v.5.1’ for imputation. Reads were aligned using the ‘Morex v3’ reference genome [39]. Data were filtered in TASSEL v5.2.58 [40] for homozygous markers with a minor allele frequency (MAF) > 0.05 and <90% missing data. A representative set of markers was selected through applying a thinning filter for a minimum distance of 0.5 Mbp, such that 2358 SNP markers were selected for use in subsequent analysis with 255–403 SNPs per chromosome. 

Relationships between genotypes were analyzed using STRUCTURE v2.3.4 software [41]. The analysis was run with an admixture model with a 100,000 burn-in period and 100,000 Markov chain Monte Carlo (MCMC) replications. The analysis was performed for K = 1–5 with 5 iterations at each K. CLUMPAK v1.1 software [42] was used to visualize clusters and to identify the most likely number of sub-populations. 

## 5. Conclusions

Our findings support the value of using historical germplasms for the identification of disease resistance in modern barley production issues, particularly Fusarium head blight. Significant variation for FHB and DON accumulation was observed in the set of European heritage varieties at levels lower than those in Canadian modern varieties. Taken together with agronomic and malt quality data, barley breeders will be able to make informed decisions when planning their new crossing schemes to develop FHB-resistant varieties. Germplasms identified within the current study could serve as valuable sources of resistance, potentially with less impediments than typically encountered when exotic material is used as a parental resistance source. Several resistant varieties were identified with a reasonable heading date and yield, including ‘Chevallier Chile’, ‘Domen’, ‘Djugay’, ‘Hannchen’, ‘Heils Franken’, ‘Moravian Barley’, and ‘Loosdorfer’, with ‘Golden Melon’, ‘Nutans Moskva’, and ‘Vellavia’ being some of the most promising varieties when malting quality characteristics were also considered.

## Figures and Tables

**Table 1:** Descriptive statistics of days to heading, Fusarium head blight rating, and deoxynivalenol content over 2018–2022.

	2018		2019			2020			2021		2022		
	Head	DON	Head	FHB	DON	Head	FHB	DON	FHB	DON	Head	FHB	DON
Mean	55.35	0.48	59.2	1.28	3.12	54.55	3.13	6.86	1.84	4.95	59.07	2.07	21.65
SE	0.74	0.10	0.37	0.10	0.49	0.64	0.12	0.75	0.13	0.69	0.41	0.10	1.52
Min	37	0.12	49	0.5	0.22	46	0.5	0.61	0	0.30	44	1	4.06
Max	60	3.33	67	5	24.39	65	5	43.67	5	32.91	69	5	84.42

Head = days from sowing to 50% heading. FHB = Fusarium head blight rating (0–5); DON = deoxynivalenol content (mg kg^−1^) as measured via enzyme linked-immunosorbent assay (ELISA). SE = standard error; Min = minimum; Max = maximum. FHB ratings and days to heading were not recorded for 2018 and 2021, respectively.

**Table 2:** Fusarium head blight scores and deoxynivalenol content for the set of European heritage barley varieties and Canadian modern check varieties.

Name	FHB Rating (0–5)		DON Log_10_ (x + 1)		Average
	(0–5)		(mg kg^−1^)		DON_Rank
**AAC Synergy**	1.9	CDEF	12.9 (0.82)	ABCDEFG	29.0
**AAC Ling**	2.1	BCDEF	10.3 (0.86)	ABCDEF	31.5
**Norman ^1^**	2.4	ABCDEF	4.6 (0.62)	BCDEFG	20.1
**AAC Goldman ^2^**	1.7	CDEF	9.4 (0.73)	ABCDEFG	26.0
Asplund	3.1	ABCD	13.1 (0.90)	ABCD	33.3
B8209	3.7	AB	15.1 (0.99)	AB	34.0
Bere	2.2	BCDEF	21.7 (1.08)	A	37.8
Bohmische-Nackte ^N^	2.9	ABCDE	10.4 (0.85)	ABCDE	30.0
Chevallier 1	1.3	EF	6.5 (0.58)	CDEFG	18.0
Chevallier Chile	1.4	EF	3.4 (0.55)	CDEFG	16.1
Chevallier French	1.6	DEF	4.7 (0.53)	CDEFG	15.2
Djugay	2.5	ABCDEF	5.4 (0.54)	CDEFG	15.9
Domen	2.0	BCDEF	2.4 (0.45)	G	10.3
Ducksbill	1.8	CDEF	11.0 (0.77)	ABCDEFG	27.3
Golden Melon	1.6	CDEF	4.2 (0.49)	EFG	11.9
Golden Pheasant	1.4	DEF	5.4 (0.62)	BCDEFG	19.9
Golden Promise	2.6	ABCDEF	8.4 (0.68)	BCDEFG	21.3
Gotlands	1.7	CDEF	5.4 (0.58)	CDEFG	15.9
Hado Streng	1.8	CDEF	7.0 (0.67)	BCDEFG	23.3
Hakata 2	1.2	EF	7.0 (0.64)	BCDEFG	20.7
Hanna	1.6	CDEF	5.9 (0.54)	CDEFG	17.1
Hannchen	2.0	BCDEF	4.7 (0.52)	DEFG	13.9
Heils-Franken	1.7	CDEF	3.3 (0.49)	EFG	11.2
Hen Gymro	2.3	ABCDEF	4.0 (0.54)	CDEFG	15.7
Isaria	2.6	ABCDEF	6.6 (0.57)	CDEFG	16.4
Ketch	2.3	ABCDEF	9.7 (0.65)	BCDEFG	20.8
Kitchin	1.1	F	5.1 (0.57)	CDEFG	17.3
Kneifel	2.1	BCDEF	10.2 (0.69)	ABCDEFG	22.8
Larker	3.3	ABC	13.6 (1.00)	AB	34.3
Long Eared Nottingham	1.4	DEF	3.9 (0.53)	DEFG	13.8
Loosdorfer	1.3	EF	3.2 (0.45)	FG	11.2
Mestny	1.8	CDEF	11.0 (0.92)	ABC	28.9
Moravian Barley	1.8	CDEF	5.2 (0.59)	CDEFG	18.9
Nurnberg	2.8	ABCDEF	10.9 (0.76)	ABCDEFG	26.9
Nutans Moskva	2.1	BCDEF	4.6 (0.55)	CDEFG	16.0
Pflugs-intensiv	2.1	BCDEF	6.4 (0.66)	BCDEFG	20.7
Prior	4.0	A	7.3 (0.79)	ABCDEFG	26.6
Proctor	2.4	ABCDEF	4.1 (0.49)	EFG	11.4
Sativum Jessen England	1.8	CDEF	8.7 (0.83)	ABCDEFG	26.7
Scotch Annat	2.7	ABCDEF	9.6 (0.76)	ABCDEFG	25.3
Scotch Common	2.5	ABCDEF	11.0 (0.81)	ABCDEFG	29.3
Vellavia	2.4	ABCDEF	4.1 (0.48)	EFG	15.1

Check varieties are presented in boldface. ^N^ Naked variety. Mean DON content (mg kg^−1^) over all years, where the value in brackets represents the mean transformed value of Log_10_ (x + 1). The following letters represents the significance group; shared letters indicate that values do not differ significantly. Moderately resistant variety, developed via in vitro selection ^1^ [15]; cross involving exotic Chinese two-row accession ‘Harbin’ ^2^ [16].

**Table 3:** Means over years for days to heading, height, days to maturity, and yield for the set of European heritage barley varieties and Canadian modern check varieties.

Name	Days to Heading		Height (cm)		Days to Maturity		Yield (kg ha^−1^)	
**AC Metcalfe**	43.9	JKLMNOP	68.6	ABCD	78.4	BCDEFG	3372	A
**CDC Copeland**	46.6	FGHIJKL	68.8	ABCDE	81.4	ABCDE	2836	ABCD
**AAC Ling**	43.0	KLMNOPQ	69.4	ABCD	79.1	BCDEFG	2792	ABCD
**AAC Synergy**	44.4	IJKLMN	65.4	ABCDE	76.6	CDEFGHI	3195	AB
Asplund	41.9	MNOPQ	65.4	ABCDE	68.9	JK	2263	ABCDE
B8209	42.9	LMNOPQ	62.9	BCDEF	72.9	GHIJK	2620	ABCDE
Bere	41.9	MNOPQ	64.6	ABCDE	77.6	CDEFGHI	2214	ABCDE
Bohmische Nackte ^N^	45.4	HIJKLMN	66.4	ABCDE	68.9	JK	1832	CDE
Chevallier 1	51.4	ABCDE	74.9	AB	82.1	ABCDE	2555	ABCDE
Chevallier Chile	43.4	KLMNOP	74.4	AB	70.9	IJK	2635	ABCD
Chevallier French	53.6	ABC	69.6	ABC	89.4	A	2346	ABCDE
Djugay	44.9	HIJKLMN	72.4	ABC	71.1	HIJK	2716	ABCD
Domen	43.9	JKLMNOP	72.9	ABC	77.4	CDEFGHI	2834	ABCD
Ducksbill	48.9	DEFGH	67.6	ABCDE	82.4	ABCDE	1813	CDE
Golden Melon	44.9	HIJKLMN	72.6	ABC	75.4	EFGHIJ	2599	ABCDE
Golden Pheasant	53.1	ABC	69.6	ABC	85.8	AB	1791	CDE
Golden Promise	51.9	ABCD	50.6	F	82.4	ABCDE	1692	DE
Gotlands	50.6	BCDEF	72.6	ABC	82.8	ABCDE	2362	ABCDE
Hado Streng	42.9	LMNOPQ	72.1	ABC	73.4	FGHIJK	2152	BCDE
Hakata 2	46.9	FGHIJKL	74.4	AB	80.1	BCDEF	2137	BCDE
Hanna	48.4	DEFGHI	71.4	ABC	78.1	BCDEFGHI	2575	ABCDE
Hannchen	47.6	EFGHIJ	68.4	ABCD	79.9	BCDEFG	2806	ABCD
Heils.Franken	45.9	GHIJKLM	69.6	ABC	78.9	BCDEFG	2784	ABCD
Hen Gymro	53.9	AB	69.4	ABC	84.1	ABCD	2231	ABCDE
Isaria	48.6	DEFGH	65.9	ABCDE	81.8	ABCDE	2819	ABCD
Ketch	46.1	GHIJKL	67.4	ABCDE	79.1	BCDEFG	2768	ABCD
Kitchin	46.9	FGHIJKL	76.6	A	77.6	CDEFGHI	2252	ABCDE
Kneifel	44.1	JKLMNO	68.1	ABCD	78.4	BCDEFG	2247	ABCDE
Larker	39.9	PQ	66.1	ABCDE	69.1	JK	2892	ABC
Long Eared Nottingham	49.6	CDEFG	73.4	ABC	84.4	ABC	2604	ABCDE
Loosdorfer	45.1	HIJKLMN	73.1	ABC	76.9	CDEFGHI	2682	ABCD
Mestny	40.1	OPQ	55.4	EF	67.6	K	2252	ABCDE
Moravian Barley	43.6	JKLMNOP	72.4	ABC	75.4	EFGHIJ	2727	ABCD
Nurnberg	44.9	HIJKLMN	76.4	A	78.1	BCDEFGH	2399	ABCDE
Nutans Moskva	43.6	JKLMNOP	68.1	ABCD	79.4	BCDEFG	3144.0	AB
Pflugs.intensiv	44.4	IJKLMN	74.6	AB	79.4	BCDEFG	2232	ABCDE
Prior	39.1	Q	61.1	CDEF	68.1	K	2499	ABCDE
Proctor	54.9	A	68.4	ABCD	84.4	ABC	2103	BCDE
Sativum Jessen England	41.4	NOPQ	56.4	DEF	78.1	BCDEFGH	1439	E
Scotch Annat	44.9	HIJKLMN	68.6	ABCD	77.4	CDEFGHI	2724	ABCD
Scotch Common	40.1	OPQ	68.9	ABCD	76.4	DEFGHI	2583	ABCDE
Vellavia	47.4	EFGHIJK	67.9	ABCDE	79.4	BCDEFG	2833	ABCD

Check varieties are presented in boldface. ^N^ Naked variety. The following letters represent significance groups, where shared letters indicate that values do not differ significantly.

**Table 4:** Means over years for physical grain characteristics for the set of European heritage barley varieties and Canadian modern check varieties.

Name	Test Weight		Thousand Kernel		Kernel Plumpness	
	(kg hL^−1^)		Weight (g)		>6/64 (%)	
**AC Metcalfe**	68.5	BCDE	48.3	ABCDEF	96.4	ABC
**CDC Copeland**	66.7	BCDEF	51.5	ABCDE	100.0	A
**AAC Ling**	67.2	BCDEF	54.6	A	98.5	ABC
**AAC Synergy**	68.3	BCDEF	50.3	ABCD	97.4	AB
Asplund	65.2	BCDEF	38.3	FG	79.1	EF
B8209	65.3	BCDEF	38.8	FG	79.3	EF
Bere	66.3	BCDEF	43.2	ABCDEFG	83.6	ABCDE
Bohmische Nackte ^N^	75.0	A	38.0	G	63.3	F
Chevallier 1	65.8	BCDEF	46.0	ABCDEFG	89.1	ABCDE
Chevallier Chile	67.5	BCDEF	41.8	EFG	85.3	BCDE
Chevallier French	68.8	ABCDE	39.5	G	82.3	DE
Djugay	64.9	CDEF	43.9	BCDEFG	89.9	ABCDE
Domen	67.9	BCDEF	45.3	ABCDEFG	95.6	ABC
Ducksbill	65.5	CDEF	46.9	ABCDEFG	95.4	ABC
Golden Melon	67.9	BCDEF	42.4	DEFG	92.5	ABCDE
Golden Pheasant	66.0	BCDEF	43.6	BCDEFG	91.4	ABCDE
Golden Promise	66.1	BCDEF	44.3	BCDEFG	93.3	ABCDE
Gotlands	69.3	ABCD	44.7	ABCDEFG	90.2	ABCDE
Hado Streng	67.3	BCDEF	44.7	ABCDEFG	90.7	ABCDE
Hakata 2	65.6	CDEF	51.0	AB	95.0	ABC
Hanna	66.9	BCDEF	47.3	ABCDEFG	90.2	ABCDE
Hannchen	68.2	BCDEF	42.5	CDEFG	86.5	ABCDE
Heils.Franken	66.4	BCDEF	50.8	ABC	94.3	ABCDE
Hen Gymro	64.5	DEF	45.6	ABCDEFG	88.3	ABCDE
Isaria	70.9	AB	43.7	BCDEFG	95.2	ABC
Ketch	69.1	ABCDE	43.4	BCDEFG	90.9	ABCDE
Kitchin	64.0	EF	50.7	ABCD	89.5	ABCDE
Kneifel	66.0	BCDEF	41.1	FG	93.1	ABCDE
Larker	63.6	CDEF	42.1	BCDEFG	96.1	ABCDE
Long Eared Nottingham	66.7	BCDEF	44.1	BCDEFG	91.4	ABCDE
Loosdorfer	67.3	BCDEF	40.1	EFG	87.6	ABCDE
Mestny	63.2	DEF	42.0	BCDEFG	97.3	ABCD
Moravian Barley	67.9	BCDEF	43.9	BCDEFG	93.7	ABCDE
Nurnberg	67.2	BCDEF	46.0	ABCDEFG	91.1	ABCDE
Nutans Moskva	69.8	ABC	43.0	BCDEFG	93.0	ABCDE
Pflugs.intensiv	66.3	BCDEF	45.4	ABCDEFG	89.0	ABCDE
Prior	64.1	EF	44.0	BCDEFG	85.9	ABCDE
Proctor	67.1	BCDEF	43.5	BCDEFG	90.7	ABCDE
Sativum Jessen England	62.0	F	41.0	BCDEFG	94.9	ABCDE
Scotch Annat	68.0	ABCDEF	48.5	ABCDEFG	86.3	ABCDE
Scotch Common	65.7	BCDEF	45.8	ABCDEFG	90.7	ABCDE
Vellavia	66.3	BCDEF	48.4	ABCDEF	84.2	CDE

Check varieties are presented in boldface. ^N^ Naked variety. The following letters represent significance groups, where shared letters indicate that values do not differ significantly.

**Table 5:** Malting quality character data for the set of European heritage barley varieties and Canadian modern varieties.

Variety	Grain		Fine Grind		Soluble		Soluble to		Alpha		Diastatic	
	Protein		Extract		Protein		Total Protein		Amylase		Power	
	(%)		(%)		(%)		(%)		(DU) ^1^		(°L) ^2^	
**AC Metcalfe**	12.9	ABC	69.0	AB	5.00	ABCD	39.1	ABC	12.1	AB	194	A
CDC Copeland *	11.7	BC	69.2	ABC	5.75	A	47.7	A	13.0	ABC	152	ABCDE
AAC Ling *	13.1	ABC	66.5	ABCDEF	4.45	CDEFG	34.0	BCDE	8.4	ABCD	107	ABCDE
**AAC Synergy**	12.0	C	70.0	A	4.85	ABCD	40.7	AB	12.4	A	165	ABCD
Bere *	12.8	ABC	62.3	CDEFG	3.85	FG	30.4	BCDE	6.0	D	75	BCDE
Bohmische-Nackte *^N^	13.0	ABC	62.6	CDEFG	4.25	DEFG	32.8	BCDE	5.9	D	147	ABCDE
Chevallier 1	12.7	BC	63.0	DEF	4.30	DEFG	34.4	BCDE	6.7	D	118	ABCDE
Chevallier Chile	13.6	ABC	63.0	CDEF	4.50	CDEFG	33.2	BCDE	7.6	D	140	ABCDE
Chevallier French	13.1	ABC	67.1	ABCD	4.50	CDEFG	34.7	BCDE	7.6	D	102	BCDE
Djugay	15.2	AB	62.6	DEF	4.05	EFG	26.6	E	7.5	D	133	ABCDE
Domen	13.7	ABC	66.1	ABCDEF	4.40	DEFG	32.0	BCDE	8.0	CD	117	ABCDE
Ducksbill	15.0	AB	63.7	CDEF	5.25	ABC	35.3	BCDE	10.4	ABCD	193	A
Golden Melon	12.7	ABC	66.7	ABCDE	4.70	CDEF	37.0	ABCD	8.5	ABCD	119	ABCDE
Golden Pheasant	14.4	ABC	64.6	BCDEF	5.50	AB	38.5	ABCD	10.2	ABCD	134	ABCDE
Golden Promise	15.5	A	63.6	CDEF	4.85	ABCD	31.4	CDE	7.7	D	192	A
Gotlands *	14.1	ABC	63.7	BCDEF	4.55	CDEFG	32.2	BCDE	7.5	ABCD	133	ABCDE
Hado Streng	15.0	AB	64.0	BCDEF	4.55	CDEFG	30.3	DE	7.5	D	111	ABCDE
Hakata 2	14.7	ABC	64.2	BCDEF	4.75	BCDEF	32.3	BCDE	8.6	ABCD	177	AB
Hanna	13.6	ABC	64.3	BCDEF	4.60	CDEFG	33.9	BCDE	7.8	D	128	ABCDE
Hannchen	13.0	ABC	65.8	ABCDEF	4.60	CDEFG	35.3	BCD	8.3	BCD	115	ABCDE
Heils-Franken	14.5	ABC	65.8	ABCDEF	4.50	CDEFG	31.0	CDE	8.7	ABCD	139	ABCDE
Hen Gymro	12.4	BC	61.8	EFG	4.50	CDEFG	36.2	BCD	7.8	D	117	ABCDE
Isaria	12.7	BC	64.7	BCDEF	4.05	EFG	32.3	BCDE	7.0	D	82	CDE
Ketch	13.3	ABC	65.7	ABCDEF	4.50	CDEFG	34.1	BCDE	7.4	D	80	DE
Kitchin	14.4	ABC	62.8	DEF	4.40	DEFG	30.7	CDE	9.1	ABCD	141	ABCDE
Kneifel	12.9	ABC	67.3	ABCD	4.75	BCDEF	36.9	BCD	8.2	BCD	112	ABCDE
Long Eared Nottingham	12.9	ABC	63.7	CDEF	4.60	CDEFG	36.0	BCD	7.5	D	99	BCDE
Loosdorfer	13.5	ABC	67.0	ABCD	4.80	BCDE	35.5	BCD	8.8	ABCD	138	ABCDE
Mestny *^P^	-		60.3	FG	3.85	FG			6.2	D	114	ABCDE
Moravian Barley	13.0	ABC	66.0	ABCDEF	4.45	DEFG	34.5	BCDE	8.3	BCD	98	BCDE
Nurnberg	15.0	AB	63.4	CDEF	4.65	CDEFG	31.2	CDE	7.9	CD	139	ABCDE
Nutans Moskva	12.8	ABC	66.4	ABCDEF	4.25	DEFG	33.2	BCDE	8.2	BCD	109	ABCDE
Pflugs-intensiv	14.6	ABC	62.6	DEF	4.80	BCDE	33.2	BCDE	8.3	BCD	133	ABCDE
Prior	14.6	ABC	63.6	CDEF	4.65	CDEFG	31.9	CDE	8.9	ABCD	146	ABCDE
Proctor	13.4	ABC	65.1	ABCDEF	4.70	CDEF	35.1	BCDE	7.0	D	133	ABCDE
Sativum Jessen England *^P^	-		56.2	G	3.75	G			6.1	D	94	ABCDE
Scotch Annat *	12.4	ABC	61.6	DEFG	3.85	FG	31.3	BCDE	6.0	D	53	E
Scotch Common *	13.0	ABC	65.9	ABCDEF	5.05	ABCD	38.4	ABCD	10.4	ABCD	187	ABC
Vellavia	13.0	ABC	66.7	ABCDE	4.60	CDEFG	35.5	BCD	9.9	ABCD	165	ABCD

^1^ Alpha amylase (dextrinizing unit of the measure of alpha amylase activity, DU); ^2^ diastatic power (degrees Lintner); ‘*’ indicates evaluation in a single year; ^N^ naked variety; ^P^ pigmented variety, where NIR was not possible due to colouration. Boldface indicates a modern variety. The following letters represent significance groups, where shared letters indicate that values do not differ significantly.

**Table 6:** List of 38 European heritage barley varieties and two-row Canadian cultivars used within the study.

Name	GRU Store Code ^1^	Pedigree ^2^	Origin ^2^	Type ^3^
AC Metcalfe	Check ^AM^	AC Oxbow/Manley	CAN (western)	2-row
AAC Synergy	Check ^AM,F^	TR02267/Newdale	CAN (western)	2-row
AAC Ling	Check ^AM,F^	Leader/Pasadena	CAN (eastern)	2-row
CDC Copeland	Check ^AM^	WM861-5/TR118	CAN (western)	2-row
Norman	Check ^F^	In vitro selection from CDC Kendall	CAN (western)	2-row
AAC Goldman	Check ^F^	TR04282/Newdale	CAN (western)	2-row
Asplund	B4534	Mixed seed lot (Primus-I/6-row land race)	SWE	6-row
B8209 ^5^	B8209			6-row
Bere	B3339	Scottish land variety	GBR: Scotland	4-row ^4^
Bohmische-Nackte ^N^	B4425	Czech land variety	CZE	6-row
Chevallier 1	B3432	English land variety	GBR	2-row
Chevallier Chile	B3431	English land variety	GBR	2-row
Chevallier French	B3437	English land variety	GBR	2-row
Djugay	B3684	USSR land variety	SUN	2-row
Domen	B3631	Opal B/Maskin	NOR	2-row
Ducksbill	B3446	English land variety	GBR	2-row
Golden Melon	B4845	Chevallier selection	GBR; JPN	2-row
Golden Pheasant	B4819	Goldthorpe/Pfaunen	GBR	2-row
Golden Promise	B4015	Maythorpe Gamma-Ray Mutant	GBR	2-row
Gotlands	B3464	Swedish land race	SWE	2-row
Hado Streng	B8244	Hado Gerste/Strengs Franken	DEU	2-row
Hakata 2	B3472	Prior/Golden Melon	JPN	2-row
Hanna	B7281	Moravian selection	CSK	2-row
Hannchen	B7158	Hanna Selection	SWE; CAN	2-row
Heils-Franken	B7993	Frankia land variety	DEU	2-row
Hen Gymro	B7055	Welsh land variety	GBR: Welsh	2-row
Isaria	B7179	Danubia/Bavaria	DEU	2-row
Ketch	B8307	Noyep/Lenta	AUS	2-row
Kitchin	B3347	Moravian/Deficiens	USA	2-row
Kneifel	B8577	Hanna selection	DEU	2-row
Larker	B8210	Titan/Kindred/3/Newal/Peatland//Montcalm	USA	6-row
Long Eared Nottingham	B3515	English land variety	GBR	2-row
Loosdorfer	B7178	Hanna selection	AUT	2-row
Mestny ^P^	B3681	USSR land variety	SUN	6-row
Moravian Barley	B3732	Moravia land variety	CZE	2-row
Nurnberg	B4212	German land variety	ESP	2-row
Nutans Moskva	B3521	USSR land variety	SUN	2-row
Pflugs-intensiv	B4842	Saarland land variety	DEU	2-row
Prior	B3567	Chevalier selection	AUS	2-row
Proctor	B4705	Kenia/Plumage Archer	GBR	2-row
Sativum Jessen England ^P^	B17759	English land variety	GBR	6-row
Scotch Annat	B4812	Scottish land variety	GBR: Scotland	2-row
Scotch Common	B3584	Chevallier selection	GBR	2-row
Vellavia	B9933	Haute-Loire land variety	FRA	2-row

^1^ GRU, Germplasm Resources Unit Store Code John Innes Centre (JIC), Norwich, UK (https://www.seedstor.ac.uk, accessed on 5 March 2024). ^2^ Pedigree and country of origin (www.vurv.cz/barley/pedigree/pedigree.php, accessed on 5 March 2024). ^3^ Barley spike morphology (two-row or six-row barley type). ^4^ Four-row barley (three grains per spikelet, where outer laterals from opposite sides of the rachis form a row). ^5^ Classified as two-row by GRU. ^AM^ Included in the study in the agronomy and malting quality test; ^F^ included in the study in the Fusarium nursery test; ^N^ naked barley; ^P^ pigmented barley.

## Data Availability

Primary data sets and genetic data are available upon request through a standard material transfer agreement.

## Supplementary Materials

The following supporting information can be downloaded at https://www.mdpi.com/article/10.3390/plants13060799/s1: Table S1. Mean Fusarium head blight ratings and DON content of eighty European barley varieties evaluated in 2017 at Charlottetown, PE, Canada (*n* = 3), Table S2. Monthly means (May–Aug) for various weather variables for Charlottetown, PE (2017) and Brandon, MB (2018–2022), Table S3. Principal component analysis. The first three principal components of genetic distance for heritage varieties, modern Canadian varieties, and FHB resistance sources, Figure S1. Hierarchical cluster relationships of heritage and modern varieties and common resistance sources constructed by neighbour joining method using 2358 single nucleotide polymorphic (SNP) markers.
