# Supplementary material for: Bringing Barley Back: Analysis of Heritage Varieties for Use as Germplasm Sources to Improve Resistance against the Most Devastating, Contemporary Disease in Canada, Fusarium Head Blight (Fusarium graminearum)
Source: Plants (Basel). 2024 Mar 11;13(6):799. doi: 10.3390/plants13060799 (PMC10974673; doi:10.3390/plants13060799)
Supplement: Supplementary file 1 [file plants-13-00799-s001.zip › plants-2889592-supplementary.pdf]

Supplementary Table S1. Mean Fusarium head blight ratings and DON content of eighty European barley varieties evaluated in 2017 at Charlottetown, PE, Canada (n=3).

		Fusarium head blight				
		**Incidence**	**Severity**	**Index**	**DON**	**DON**
**Entry**	**Name**	**(0–10)**	**(0–10)**	**(0–100)**	**(mg kg^{-1})**	**Rank**
1	Ankara Tobak	8.0	5.3	42.7	36.7	60
2	Bere	8.0	5.3	42.7	28.9	41
3	Kitchin	7.0	4.3	30.3	18.6	22
4	Bavaria	8.0	4.3	34.7	36.4	59
5	Arabische	7.0	4.3	30.3	34.2	53
6	Burtons Malting	7.0	3.7	25.7	42.3	65
7	Beavans Archer	7.0	4.3	30.3	47.0	69
8	Chevallier Chile	8.0	4.0	32.0	16.6	14
9	Chevallier 1	5.3	4.0	21.3	57.0	77
10	Chevallier French	8.0	4.3	34.7	17.8	20
11	Ducksbill	8.0	5.7	45.3	18.6	21
12	Gotlands	5.7	4.0	22.7	27.2	35
13	Goldthorpe	6.7	4.3	28.7	33.6	50
14	Guld	8.0	5.3	42.7	34.4	54
15	Hakata 2	5.7	3.7	20.7	11.6	4
16	Kherson	7.3	4.0	29.3	22.1	28
17	Long Eared Nottingham	8.0	4.3	34.7	17.1	18
18	Nutans Moskva	8.0	5.3	42.7	8.0	1
19	Odessa	6.7	4.3	28.7	35.6	57
20	Old Wiltshire	2.7	1.3	3.3	50.5	72
21	Prior	8.0	5.3	42.7	15.0	10
22	Spratt	8.0	5.7	45.3	55.7	75
23	Scotch Common	8.0	4.3	34.7	25.7	30
24	Swanneck	8.0	5.3	42.7	68.5	79
25	Domen	6.3	4.0	25.3	14.4	9
26	Mestny	6.3	4.0	25.3	17.0	17
27	Djugay	5.3	3.0	16.0	13.7	8
28	Nikaku Chevallier	6.7	4.0	26.7	31.3	46
29	Moravian Barley	8.0	4.3	34.7	17.2	19
30	Golden Promise	6.7	4.3	28.7	26.5	32
31	Nurnberg	8.0	4.3	34.7	27.2	34
32	Hansen 181	6.3	4.0	25.3	38.3	62
33	Kavkazsky Nahy	7.7	4.0	30.7	19.6	23
34	Bohmische-Nackte	8.0	4.0	32.0	23.9	29
35	Asplund	8.0	4.3	34.7	28.1	38
36	Kagelkorn	7.7	5.0	38.3	34.9	55
37	Tamparkorn	8.0	5.3	42.7	39.3	63
38	Proctor	8.0	5.3	42.7	28.6	39
39	Scotch Annat	8.0	5.0	40.0	15.7	12
40	Old Irish	6.7	4.0	26.7	35.6	58
41	Cornish	6.0	3.7	22.0	34.1	52

42	Chevallier D10	8.0	4.3	34.7	55.8	76
43	Golden Pheasant	8.0	5.7	45.3	28.7	40
44	St Davids	7.7	4.3	33.0	38.1	61
45	Old Cromarty	8.0	5.3	42.7	53.3	74
46	Pflugs-intensiv	7.7	5.0	38.3	20.7	25
47	Golden Melon	8.0	5.3	42.7	13.4	7
48	Chevallier	7.7	4.0	30.7	87.7	80
49	Kober	6.0	3.3	20.0	52.0	73
50	Binder	6.7	4.0	26.7	46.6	68
51	Tiree 6 row	6.7	4.3	28.7	35.6	56
52	Northumberland Rogue	8.0	5.3	42.7	50.1	71
53	Hen Gymro	5.0	3.0	15.0	21.7	26
54	Hannchen	7.0	4.3	30.3	17.0	16
55	Loosdorfer	8.0	4.3	34.7	12.2	5
56	Isaria	7.7	4.0	30.7	13.2	6
57	Gull	6.0	4.0	24.0	33.7	51
58	Svanhals	8.0	5.3	42.7	42.4	66
59	Hanna	8.0	5.0	40.0	30.2	43
60	Monte Cristo	8.0	5.3	42.7	45.7	67
61	Chevron	7.7	4.0	30.7	39.3	64
62	Maythorpe	7.7	4.3	33.0	47.2	70
63	Chevallier	8.0	4.0	32.0	21.8	27
64	Heils-Franken	7.0	4.0	28.0	27.7	36
65	Betzes	5.7	3.0	17.0	30.7	44
66	Manchuria	8.0	5.3	42.7	31.2	45
67	Klages	7.0	4.0	28.0	16.7	15
68	Larker	7.7	5.3	40.7	15.8	13
69	Hado Streng	6.3	4.0	25.3	20.4	24
70	Ketch	5.3	4.0	21.3	28.0	37
71	Austrian Early	7.7	4.0	30.7	32.0	48
72	Oderbrucker	7.7	4.3	33.0	26.1	31
73	Kneifel	8.0	4.0	32.0	15.4	11
74	Arabische2	7.3	4.0	29.3	32.3	49
75	Hatif de Grignon	8.7	5.7	49.0	30.0	42
76	Glorie du Velay	5.7	2.3	13.0	10.2	2
77	Vellavia	6.3	4.0	25.3	32.0	47
78	Riojana	7.7	6.0	46.0	65.9	78
79	Sativum Jessen England	7.3	4.0	29.3	10.7	3
80	PEI - Island	6.7	4.0	26.7	26.6	33
	Grand mean	7.2	4.4	32.2	30.8	
	LSD (0.05)	0.6812	0.6905	5.278	24.2	
	F prob	<.001	<.001	<.001	<.001	
	Reps	3	3	3	3	

Supplementary Table S2. Monthly means (May-Aug) for various weather variables for Charlottetown, PE (2017) and Brandon, MB (2018-2022).

Month	Location	Year	Max Temp (°C)	Min Temp (°C)	Mean Temp (°C)	Heat Deg. Days	Cool Deg. Days	Total Precip (mm)	Dir of Max Gust (10's deg)	Speed of Max Gust (km/h)
May	Charlottetown, PE	2017	14.3	4.6	9.5	8.5	0.0	5.2	16.2	48.0
	Brandon, MB	2018	22.9	5.4	14.2	4.6	0.8	0.6	19.7	44.4
	Brandon, MB	2019	17.0	1.6	9.3	8.9	0.1	1.3	13.3	44.8
	Brandon, MB	2020	17.1	3.7	10.4	7.7	0.1	0.3	18.3	44.5
	Brandon, MB	2021	17.7	2.1	9.9	8.2	0.1	0.8	19.0	45.3
	Brandon, MB	2022	15.6	4.7	10.2	7.8	0.0	3.3	17.0	49.2
June	Charlottetown, PE	2017	20.5	10.5	15.5	3.1	0.6	3.4	18.6	43.3
	Brandon, MB	2018	25.9	12.3	19.1	1.5	3.4	4.0	20.6	45.4
	Brandon, MB	2019	23.6	9.4	16.5	2.2	0.7	2.5	19.6	42.9
	Brandon, MB	2020	25.0	10.4	17.7	1.8	1.5	7.1	22.0	52.0
	Brandon, MB	2021	26.0	11.6	18.8	0.8	1.7	3.4	24.1	46.1
	Brandon, MB	2022	22.9	10.4	16.6	2.3	1.0	2.3	22.4	47.2
July	Charlottetown, PE	2017	23.4	13.7	18.6	0.9	1.5	2.5	20.9	37.3
	Brandon, MB	2018	26.4	12.0	19.3	1.2	3.6	2.9	24.2	42.2
	Brandon, MB	2019	25.9	12.7	19.3	0.4	1.7	1.3	23.7	46.4
	Brandon, MB	2020	26.3	14.0	20.2	0.2	2.3	1.8	25.5	47.4
	Brandon, MB	2021	28.0	12.8	20.4	0.5	2.9	0.0	22.1	37.8
	Brandon, MB	2022	25.4	13.7	19.5	0.4	1.9	2.5	25.0	45.3
August	Charlottetown, PE	2017	23.3	13.5	18.4	0.9	1.3	4.4	19.2	35.2
	Brandon, MB	2018	26.8	9.4	18.1	3.5	3.4	1.5	23.2	40.3
	Brandon, MB	2019	23.9	9.7	16.8	2.0	0.8	2.4	23.0	41.4
	Brandon, MB	2020	26.0	11.8	18.9	0.5	1.4	2.0	28.7	44.3
	Brandon, MB	2021	23.9	11.1	17.5	3.8	2.8	10.1	24.4	45.8
	Brandon, MB	2022	26.0	12.5	19.2	0.4	1.7	0.9	22.4	44.8

Source: https://climate.weather.gc.ca/historical_data/search_historic_data_e.html
Max Temp = highest temperature in degrees Celsius (°C) observed at the location; Min Temp = lowest temperature in degrees Celsius (°C) observed at the location; Mean Temp = average of the maximum and minimum temperature at the location; Heat Deg. Days = number of degrees Celsius that the mean temperature is below 18 °C; Cool Deg. Days = number of degrees Celsius that the mean temperature is above 18 °C; Total precip = sum of the total rainfall in millimetres; Dir of Max Gust = direction of the maximum gust from which the wind blows, expressed in tens of degrees; Speed of Max Gust = speed in kilometres per hour of the maximum wind gust during the day.

Supplementary Table S3. Principal component analysis. The first three principal components of genetic distance for heritage varieties, modern Canadian varieties, and FHB resistance sources.

Variety	PC1	PC2	PC3
AAC_Synergy	18.317	-0.488	1.715
AAC_Goldman	17.387	-1.081	2.473
TR04282	17.040	-0.540	4.163
TR253	16.175	-1.303	0.407
AC_Oxbow	15.824	-1.023	0.537
AC_Metcalfe	14.486	-1.716	0.351
CDC_Mindon	14.396	-0.926	1.321
Conlon	10.510	2.863	-1.037
Norman	10.348	-3.628	0.872
Djugay	3.686	6.833	-3.042
Vellavia	2.073	-3.136	-0.874
AAC_Ling	1.651	-3.809	-1.125
Ducksbill	-0.157	0.563	9.630
GB132013	-0.285	3.039	-3.538
Kitchin	-0.402	5.347	-4.075
Mestny	-0.901	8.639	-2.294
CI_4196	-0.973	-1.970	7.930
Chevron	-1.115	15.976	-1.374
Chevallier_French	-1.207	-4.555	2.660
Sativum_Jessen_England	-1.208	3.578	-3.347
Isaria	-1.282	-4.908	-3.093
Ketch	-1.296	-6.578	-2.921
Nutans_Moskva	-1.436	-7.056	-5.351
Proctor	-1.477	-5.917	1.114
Bere	-1.484	-7.118	-5.367
B8209	-1.582	11.230	-7.150
Svanhals	-1.687	-4.490	-4.320
Larker	-1.714	14.286	-0.831
Island	-2.096	-3.435	-4.109
Bohmische-Nackte	-2.711	13.134	-3.497
Golden_Promise	-3.031	-5.266	4.243
Loosdorfer	-3.187	20.387	-2.035
Asplund	-3.201	20.451	-2.006
Hannchen	-3.331	-4.796	-4.908
Heils-Franken	-3.443	-3.755	-3.314
Domen	-3.598	-5.320	-6.831
Kutahya	-3.872	1.794	1.716
Moravian_Barley	-3.915	-5.667	-1.908
Hen_Gymro	-4.124	-1.929	-2.843
Golden_Melon	-4.169	-4.903	-6.503

Prior	-4.822	1.814	1.856
Chevallier	-4.977	-4.488	-1.280
Scotch_Annat	-5.007	-5.792	-1.004
Nurnberg	-5.165	-5.419	-4.347
Hanna	-5.242	-3.158	-2.394
Hakata_2	-5.304	0.742	-2.457
Chevallier_Chile	-5.466	-1.431	-3.877
Harbin	-5.634	3.615	12.043
Kneifel	-5.809	-3.847	-3.478
Hado_Streng	-5.973	-2.589	-5.376
Pflugs-intensiv	-6.068	-2.575	-3.366
Golden_Pheasant	-6.128	-3.859	2.807
Frederickson	-6.479	0.520	17.028
Zhedar_1	-6.598	0.383	16.949
Russian_6	-6.679	0.482	17.043
Long_Eared_Nottingham	-6.962	-3.567	0.045
Gotlands	-7.687	-2.961	7.407

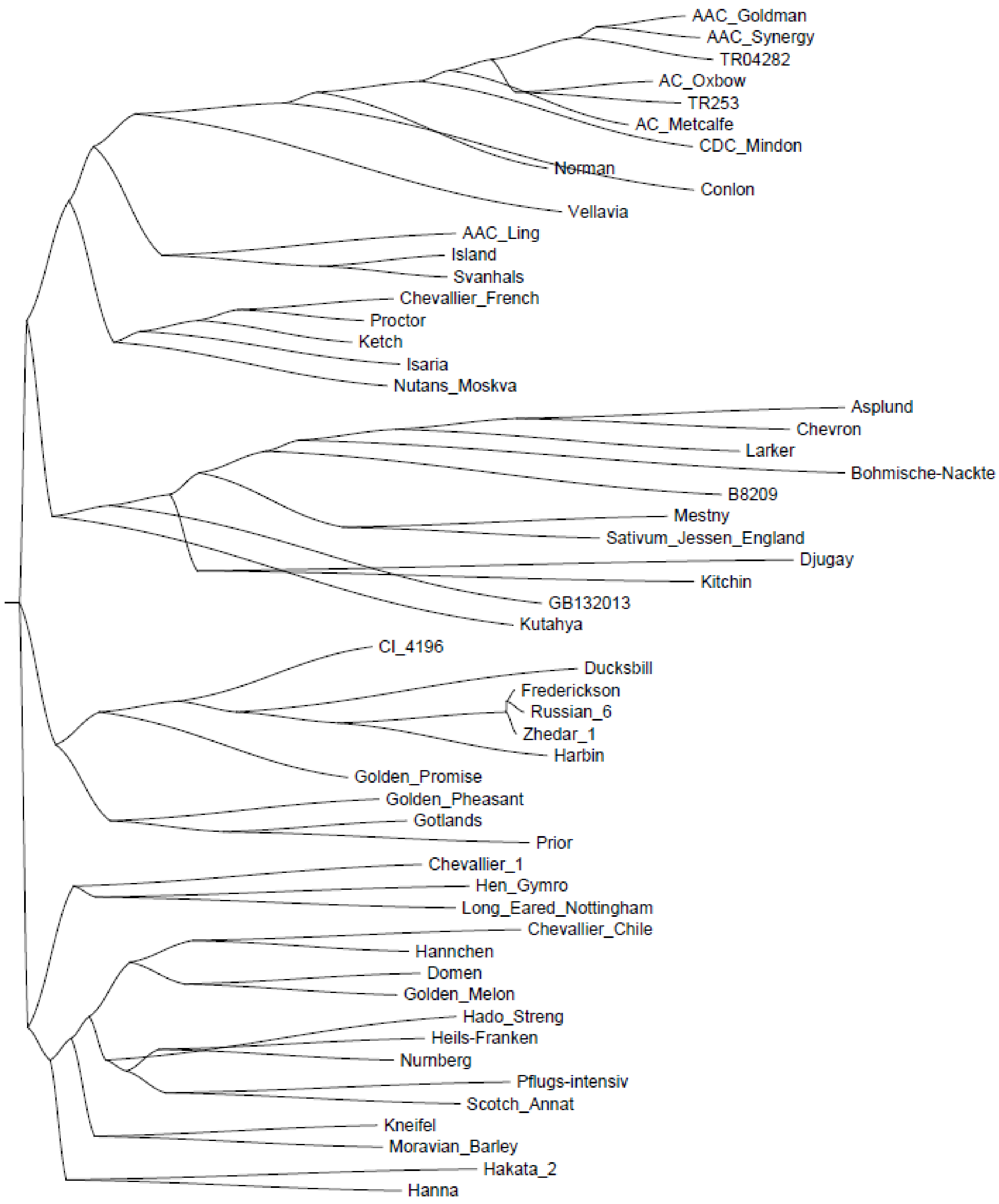

Supplementary Figure S1. Hierarchical cluster relationships of heritage and modern varieties and common resistance sources constructed by neighbour joining method using 2,358 single nucleotide polymorphic (SNP) markers.
